# The Anti-Cancer IgM Monoclonal Antibody PAT-SM6 Binds with High Avidity to the Unfolded Protein Response Regulator GRP78

**DOI:** 10.1371/journal.pone.0044927

**Published:** 2012-09-19

**Authors:** Zachary Rosenes, Terrence D. Mulhern, Danny M. Hatters, Leodevico L. Ilag, Barbara E. Power, Chris Hosking, Frank Hensel, Geoffrey J. Howlett, Yee-Foong Mok

**Affiliations:** 1 Department of Biochemistry and Molecular Biology, Bio21 Molecular Science and Biotechnology Institute, University of Melbourne, Parkville, Victoria, Australia; 2 Patrys Ltd, Melbourne, Victoria, Australia; 3 Patrys GmbH, Würzburg, Germany; Duke University Medical Center, United States of America

## Abstract

The monoclonal IgM antibody PAT-SM6 derived from human tumours induces apoptosis in tumour cells and is considered a potential anti-cancer agent. A primary target for PAT-SM6 is the unfolded protein response regulator GRP78, over-expressed externally on the cell surface of tumour cells. Small angle X-ray scattering (SAXS) studies of human GRP78 showed a two-domain dumbbell-shaped monomer, while SAXS analysis of PAT-SM6 revealed a saucer-shaped structure accommodating five-fold symmetry, consistent with previous studies of related proteins. Sedimentation velocity analysis of GRP78 and PAT-SM6 mixtures indicated weak complex formation characterized by dissociation constants in the high micromolar concentration range. In contrast, enzyme-linked immunosorbant assays (ELISAs) showed strong and specific interactions between PAT-SM6 and immobilized GRP78. The apparent binding constant estimated from a PAT-SM6 saturation curve correlated strongly with the concentration of GRP78 used to coat the microtiter tray. Experiments using polyclonal antiGRP78 IgG antibodies or a monoclonal IgG derivative of PAT-SM6 did not show a similar dependence. Competition experiments with soluble GRP78 indicated more effective inhibition of PAT-SM6 binding at low GRP78 coating concentrations. These observations suggest an avidity-based binding mechanism that depends on the multi-point attachment of PAT-SM6 to GRP78 clustered on the surface of the tray. Analysis of ELISA data at high GRP78 coating concentrations yielded an apparent dissociation constant of approximately 4 nM. We propose that the biological action of PAT-SM6 in tumour cell apoptosis may depend on the multivalent nature of PAT-SM6 and the high avidity of its interaction with multiple GRP78 molecules clustered on the tumour cell surface.

## Introduction

Natural IgM antibodies play an important role in the innate immune response where they are involved in the early detection of foreign particles as well as the detection of modified self-structures including chemically modified proteins and amyloid fibrils [1], [2], [3]. IgM antibodies also participate in the recognition and removal of transformed cells as an important defence against cancer [4]. The recent development of human hybridoma technology [5] has led to the isolation of a large number of monoclonal antibodies of the IgM class from the tumours of cancer patients [6]. A number of these antibodies specifically kill malignant cells by inducing apoptotic pathways [7], highlighting the potential use of monoclonal IgM antibodies in the development of new anti-cancer treatments.

The human IgM monoclonal antibody, PAT-SM6, induces the death of tumour cells via an apoptotic pathway accompanied by intracellular lipid accumulation [8]. PAT-SM6 targets tumour cells, by binding to the protein GRP78 which is over-expressed externally on the cell surface of tumour cells [9]. GRP78, also known as BiP (immunoglobulin heavy-chain binding protein), is a member of the heat-shock protein 70 (HSP70) family that prevents stress-induced apoptosis. PAT-SM6 also binds low density lipoprotein (LDL) and oxidized LDL [8] leading to a working model for the tumour-specific apoptotic activity of PAT-SM6 whereby PAT-SM6 delivers excess lipid in the form of bound LDL or oxidized LDL into tumours by binding to modified GRP78 present on the surface of tumour cells [8].

**Figure 1:**
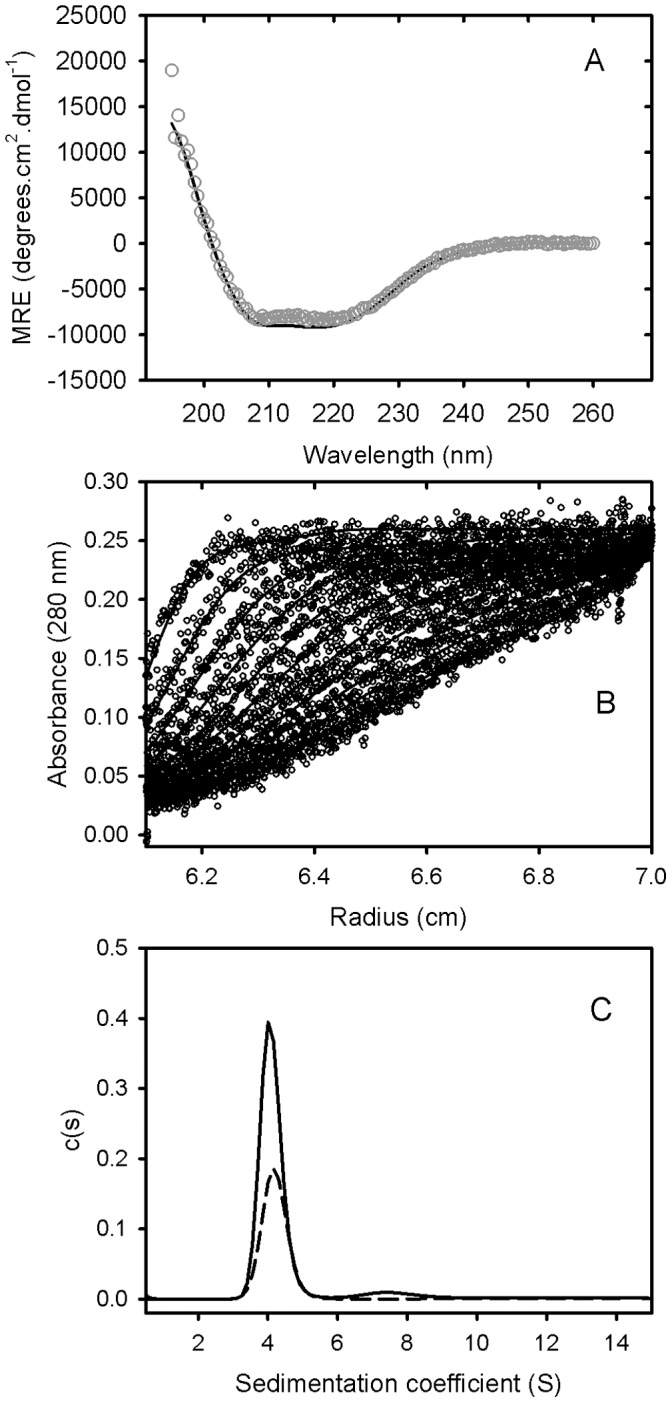
Solution characterisation of GRP78. (A) GRP78 was expressed and purified from *E.coli* cells, and subjected to circular dichroism spectroscopy. A representative spectrum for 0.15 mg/mL of purified GRP78 at 25°C (open circles) and the fit from Dichroweb using filter set sp175 (solid line) is shown. (B) Sedimentation velocity analysis of GRP78 was performed by centrifuging 0.4 mg/mL of GRP78 at 28,000 rpm in an analytical ultracentrifuge. The resulting sedimenting absorbance boundaries were monitored at 280 nm and representative data shown as open circles. Fits to the experimental data using a c(S) sedimentation model are shown as solid lines. For clarity, only every 10th scan is shown. (C) Size distribution plots for 1.2 mg/mL of GRP78 (solid line) or 0.6 mg/mL of GRP78 (dashed line) calculated from fitting data represented in (B) to a c(S) sedimentation model.

Pre-clinical models of human cancer show PAT-SM6 inhibition of tumour growth [8], suggesting a potential therapy to treat cancer. The safety and tolerability of PAT-SM6 as an anti-cancer antibody for the treatment of melanoma has been established in a recent Phase I clinical trial [10]. The further development of PAT-SM6 as an effective anti-cancer agent will be assisted by more detailed information on the structural basis and strength of the interactions of PAT-SM6 with target antigens. This knowledge is essential for the informed prediction of unwanted side effects associated with the therapeutic use of PAT-SM6 alone, or in combination therapies with other agents. In the present study we have investigated the structure and interactions of purified PAT-SM6 with recombinant human GRP78 expressed and purified from bacteria. Using sedimentation velocity analysis and enzyme-linked immunosorbent assays (ELISAs) we show that, while PAT-SM6 has a relatively low affinity for individual GRP78 molecules, the interaction of PAT-SM6 with GRP78 molecules clustered on the surface of a microtiter plate is much stronger and characterized by apparent avidity constants in the low nanomolar concentration range.

## Materials and Methods

The human monoclonal antibody PAT-SM6 and a modified hexameric derivative, PAT-SM6-hex, lacking a joining J chain, were expressed and purified from stable suspension cultures of a human cell line in serum-free media [11], [12]. Similar procedures were used to express and purify an IgG derivative (PAT-SM6 IgG) composed of the heavy and light chain sequences of PAT-SM6. Isotype control IgM was obtained from Jackson ImmunoResearch Labs, inc, West Grove, PA. The coding sequence for the full length, mature human GRP78 gene was inserted into a pPOW heat-induction vector, resulting in a construct with an N-terminal pelB secretion sequence and C-terminal 6xHis-tag [13]. The protein was expressed in *E. coli* BL21(DE3) cells and then purified from the soluble portion of cell lysate by nickel affinity chromatography using a 5 mL His-Trap column (GE Healthcare) according to the manufacturer’s protocol. Glucose isomerase was obtained from Hampton Research, Aliso Viejo, CA.

**Table 1:** Sedimentation velocity and SAXS analysis of GRP78 and PAT-SM6.^a^

Parameter	GRP78	PAT-SM6
S_20, w_	4.21	17.2
f/f_0_	1.4	1.9
MW	71500	912000
a (nm)	5.92	18.15
b (nm)	0.97	1.21
*R_g_* (Å)	45.5±2.2	124.0±2.1
*D_max_* (Å)	134	422

a Sedimentation coefficients (S_20, w_) and frictional ratios (f/f_0_) were calculated from fitting experimental sedimentation velocity data to a continuous size distribution c(s) model. These values were used to calculate the molecular weight (MW) and to model the protein shape as an oblate ellipsoid with axial dimensions a (nm) and b (nm). SAXS data for GRP78 and PAT-SM6 (Figures 2 and 4, respectively) was analysed by Guinier analysis to estimate radius of gyration, *R_g_* and *P*(*r*) analysis to estimate maximum dimension, *D_max_*.

### Circular Dichroism (CD) Measurements

CD spectra for GRP78 (0.15 mg/ml) and PAT-SM6 (0.15 mg/ml) in phosphate buffered saline (PBS; 20 mM sodium phosphate, 140 mM NaCl, pH 7.4) were acquired using an Aviv Model 62 DS CD spectrometer (Aviv Biomedical, Inc, Lakewood, New Jersey) at 25°C with a 1 mm pathlength quartz cuvette, a spectral bandwidth of 1 nm, a signal averaging time of 2 s and a data interval of 0.5 nm. Ellipticities in millidegrees were corrected for measurements of buffer alone and converted to mean residue ellipticities (MRE) using the formula MRE = ellipticity × MRW/(10 ×c ×l) where MRW is the mean residue weight in g/mol, c the concentration in mg/mL, and l the path length in cm. CD spectra were analysed to obtain estimates of secondary structure using Dichroweb [14].

**Figure 2:**
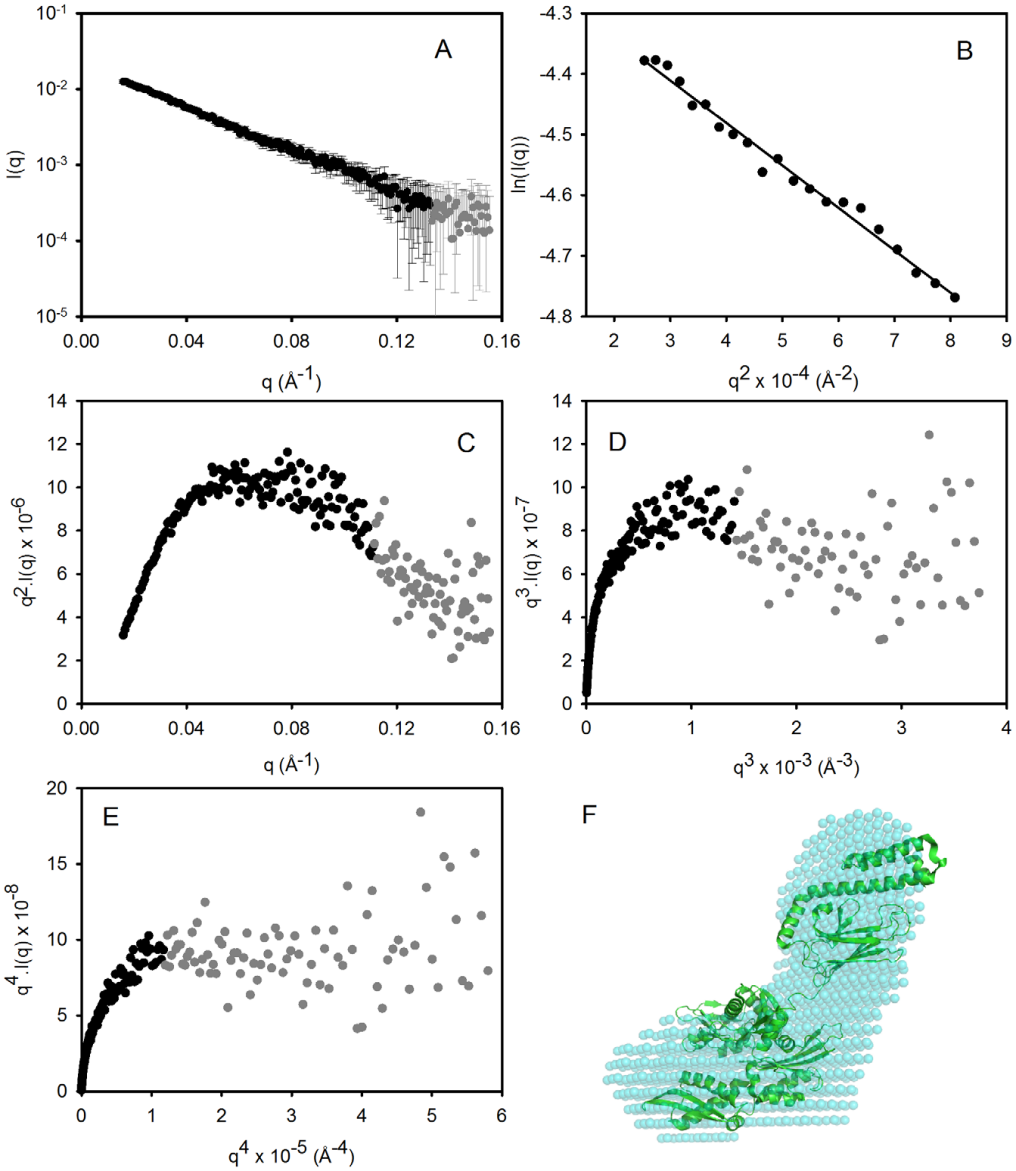
SAXS analysis of GRP78. (A) The raw SAXS data are shown as circles representing mean intensity *I*(*q*) as a function of momentum transfer *q*. Error bars indicate ±1 standard deviation (σ). Black symbols: *I*(*q*) ≥ σ and grey symbols: *I*(*q*) < σ. (B) Guinier plot for these SAXS data for q.Rg<1.3. (C) Kratky plot of the SAXS data. (D & E) Porod-Debye plots as a function of *q* ^3^ and *q* ^4^, respectively. (F) The SAXS-derived *ab initio* shape reconstruction (average filtered shape from 10 reconstructions) is shown superimposed on the NMR-refined domain arrangement of the crystallographic structures of the N-and C-terminal domains of the bacterial GRP78 homologue DnaK [26].

### Small-angle x-ray Scattering (SAXS)

SAXS data were collected at the Australian Synchrotron SAXS/WAXS beamline in conjunction with in-line gel filtration chromatography, as described by Gunn and co-workers [15]. Loading concentrations for the in-line gel filtration column was 5 mg/mL for SM6 and 5 mg/mL for GRP78. Detector images were analysed as averages of ten sequential 2 s exposures using the SAXS15ID software (Australian Synchrotron), which were then converted to individual *I*(*q*) SAXS profiles. *I*(*q*) is the scattered X-ray intensity as a function of the magnitude of the momentum transfer vector *q* = (4πsinθ)/λ, where the scattering angle is 2θ and the X-ray wavelength is λ (1.0332 Å). The *q* range over which intensities were collected was 0.0047–0.2807 Å^−1^. SAXS profiles were analysed using the ATSAS (version 2.4) suite of programs [16]. Guinier analysis was performed using AUTORG. The intensity at zero angle *I*(0) and the maximum dimension (*D_max_*) of scattering particles were estimated from *P*(*r*) pair distance vector distribution functions using AUTOGNOM. The volumes of the respective scattering particles were calculated from the area under Kratky plots (*q* ^2^.*I*(q)/*I*(0) vs. *q*) as described by Svergun and Fegin [17]; whereby, Kratky plots were extrapolated at low *q* (*q* < *q*_min_) using the Guinier approximation and extrapolated at high *q* (*q* where *I*(*q*)*/I*(0) <0.01) using the Porod approximation [18]. *Ab initio* shape reconstructions from scattering data were performed using DAMMIF [19] and averaged filtered shapes calculated from ensembles of 10 reconstructions using the DAMAVER suite of programs [20]. Rigid body refinement with the addition of missing segments was performed using CORAL [21] for multiple chain models. Theoretical scattering profiles from structural models were generated and compared with experimental SAXS data using CRYSOL [22]. Structural models and shape envelopes where optimally superimposed using SUPCOMB [23]. Statistical comparisons of the goodness of fit of theoretical scattering profiles of structural models to experimental SAXS data were conducted by calculation the *F*-statistic for pairs of fits and integrating the appropriate *F*-distribution [24].

### Sedimentation Velocity Analysis

Sedimentation velocity experiments were conducted using an XL-I analytical ultracentrifuge (Beckman Coulter, Fullerton, CA) equipped with an An-60 Ti rotor at 20°C. Protein samples were added to double-sector epon-filled centrepieces with 20 mM sodium phosphate, pH 7.0, 100 mM NaCl, 55 mM arginine in the reference compartment. Radial absorbance data were acquired at a rotor speed of 28,000 rpm, using a wavelength of 280 nm, and with radial increments of 0.003 cm in continuous scanning mode. The sedimenting boundaries were fitted to a model assuming a distribution of sedimentation coefficients for non-interacting species, c(S), using the program SEDFIT [25]. Data were fitted using a regularization parameter of p = 0.95 and floating the frictional ratio.

**Figure 3:**
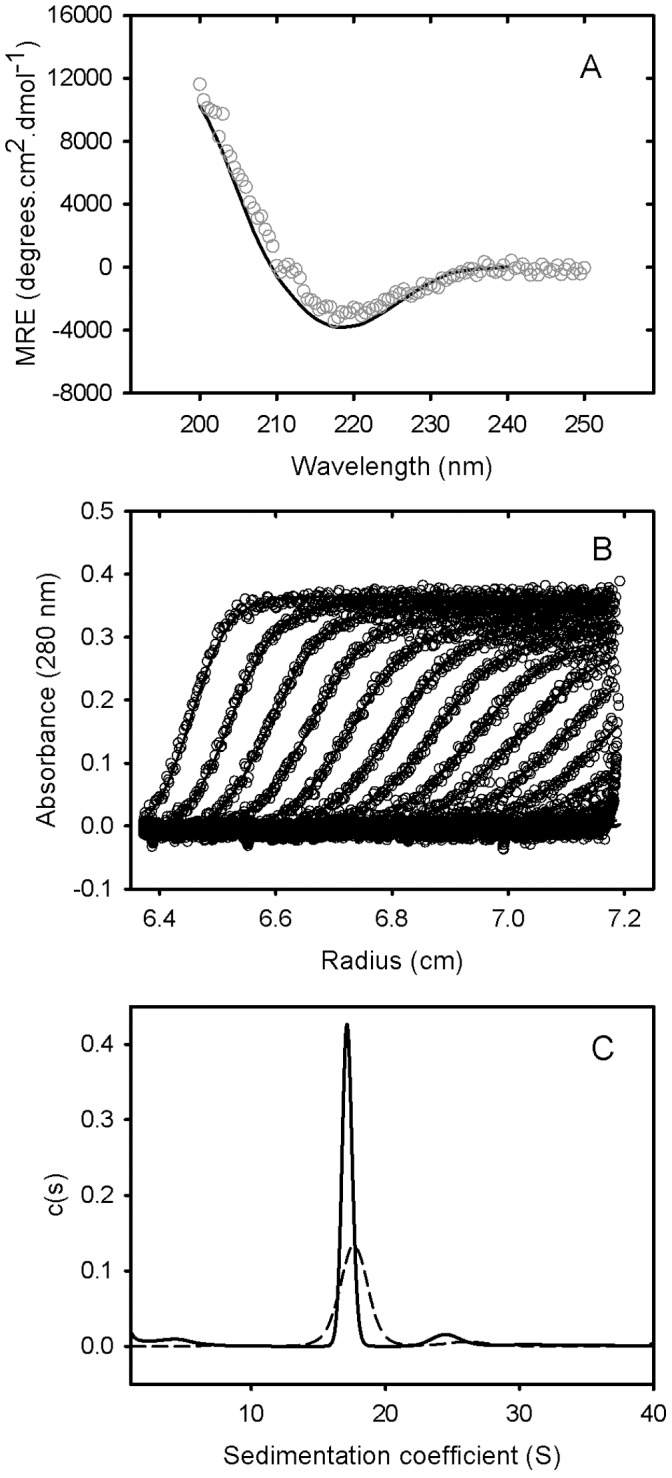
Solution characterisation of PAT-SM6. (A) 0.15 mg/mL of PAT-SM6 was subjected to circular dichroism spectroscopy. A representative spectrum at 25°C (open circles) and the fit from Dichroweb using filter set sp175 (solid line) is shown. (B) Sedimentation velocity analysis of PAT-SM6 was performed by centrifuging 0.4 mg/mL of PAT-SM6 at 28,000 rpm in an analytical ultracentrifuge. The resulting sedimenting absorbance boundaries were monitored at 280 nm and representative data shown as open circles. Fits to the experimental data using a c(S) sedimentation model are shown as solid lines. For clarity, only every 3rd scan is shown. (C) Size distribution plots for pentameric PAT-SM6 (solid line) and hexameric PAT-SM6 (dashed line) calculated from fitting data represented in (B) to a c(S) sedimentation model.

### Enzyme-linked Immunosorbant Assay (ELISA)

Antigens were coated onto 96-well microtitre trays (nunc maxisorp) by incubation overnight at 4°C. Plates were then washed 3 times in PBS, 3 times in PBS +0.1% Tween 20, 3 times in PBS and blocked with 5% (w/v) BSA in PBS for 1 hour at room temperature, then washed again as above. All antibody preparations were made in 5% (w/v) BSA in PBS. For indirect ELISA experiments, antibodies were applied directly to the plate and incubated for 1 hour. For competitive ELISAs, antibodies were incubated in solution with various amounts of antigen prior to application to the microtitre plate. After washing again as above, peroxidise-conjugated secondary antibody (goat anti-rabbit IgG (Calbiochem), rabbit anti-human IgG (Calbiochem), or rabbit anti-human IgM (Dako), dependent on the primary antibody source and class) was applied to the plate and incubated for 1 hour according to the manufacturer’s instructions. After a final wash, antibody binding was determined using 100 µL per well of 3,3′,5,5′-Tetramethylbenzidine substrate solution (Sigma) and measuring colour change at 655 nm. The time course for colour development was essentially linear over the optical density range 0–3. Measurements were taken 15 min after the addition of substrate. In control experiments, involving direct coating of the plates with PAT-SM6, over the concentration range 0–2.5 µg/ml, a systematic increase was observed in the ELISA signal with increased coating concentration, implying a direct correlation between the ELISA signal and the amount of antibody bound to the plate. ELISA data for the titration of primary antibody binding to immobilized GRP78 was analysed assuming a simple Langmuir binding isotherm described by the relationship Y = K_a_/(1−K_a_) where Y is the magnitude of the ELISA signal and K_a_ is the apparent binding constant assuming a single class of binding sites.

**Figure 4:**
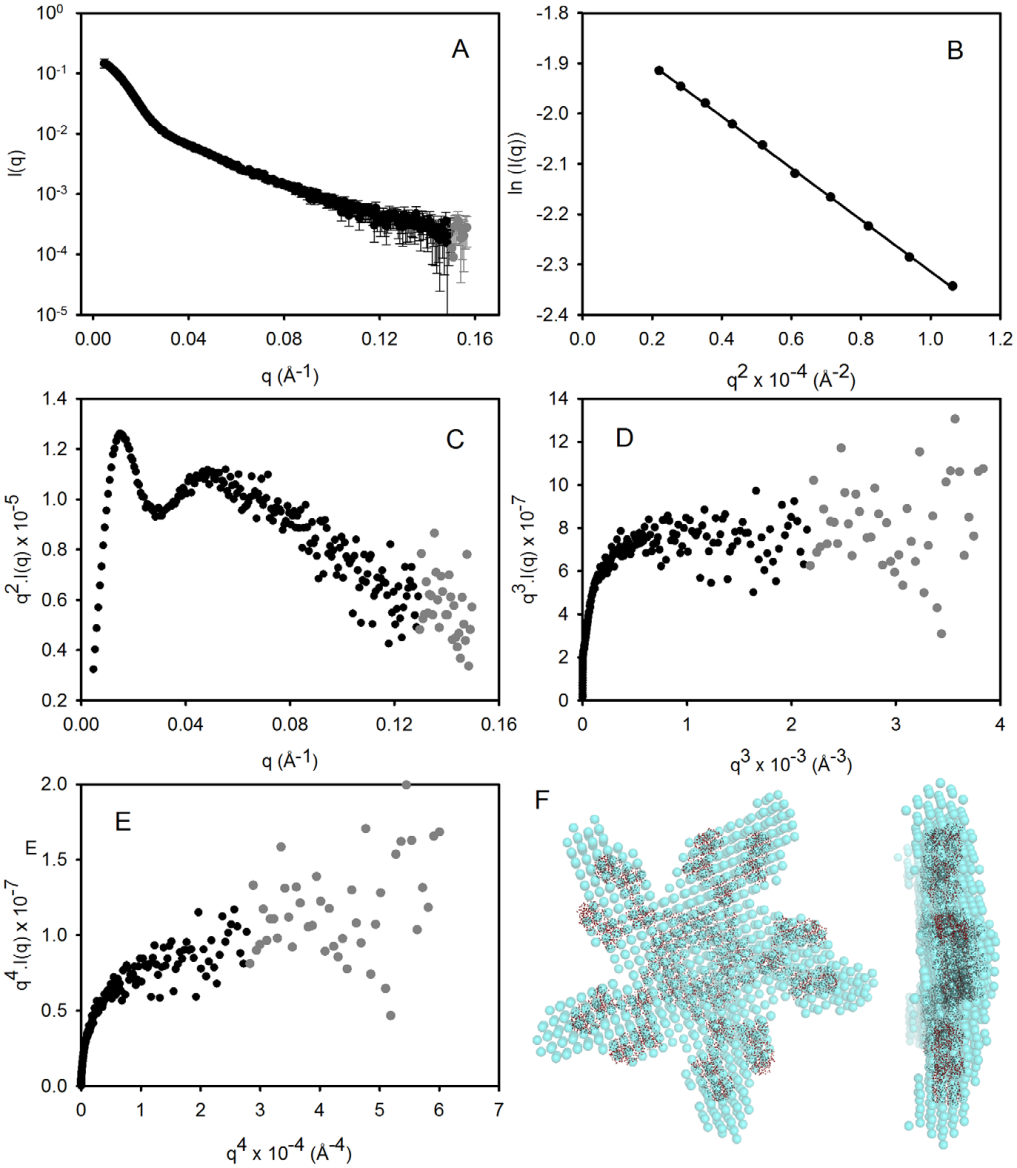
SAXS analysis of PAT-SM6. (A) The raw SAXS data are shown as circles representing mean intensity *I*(*q*) as a function of momentum transfer *q*. Error bars indicate ±1 standard deviation (σ). Black symbols: *I*(*q*) ≥ σ and grey symbols: *I*(*q*) < σ. (B) Guinier plot for these SAXS data for *q*.*R_g_*<1.3. (C) Kratky plot of the SAXS data. (D & E) Porod-Debye plots as a function of *q* ^3^ and *q* ^4^, respectively. (F) The *ab initio* shape reconstruction (average filtered shape from 10 reconstructions with explicit P5 symmetry) is shown superimposed on the rigid body refined SAXS model, generated using Fab and Fc fragments joined by a flexible 8-residue poly-Gly linker. The orientation of the four constant Ig domains of each Fc fragments was modelled on the crystal structure of the IgE and the relative Fc orientation was constrained by distance constraints enforcing inter-chain disulphide bonds [33].

## Results

### Characterisation of Purified GRP78

Human GRP78, linked to a pelB N-terminal extension and His-Tag [11] was expressed and purified from *E. coli*. Mass spectrometry of the purified product yielded a mass of 73270.7 Da compared to a theoretical value based on composition of 73269.9 Da. CD spectrophotometry indicated a folded structure with a CD spectra characterised by a double minima at approximately 208 and 221 nm (Figure 1). Analysis of the spectra yielded estimates of 22.1% and 29.1% for á and â –structure, respectively. These values may be compared with estimates of 36% á-helix and 27% â-sheet for the secondary structure determined from the three-dimensional structure of the bacterial HSP70 homologue, DnaK [26]. Additional evidence for the correct folding of purified human GRP78 was obtained by assaying of the residual ATPase activity. Use of a coupled pyruvate kinase-lactate dehydrogenase assay [27] indicated a specific activity of approximately 4 nmole ATP hydrolyzed per sec per µmole of GRP78.

**Figure 5:**
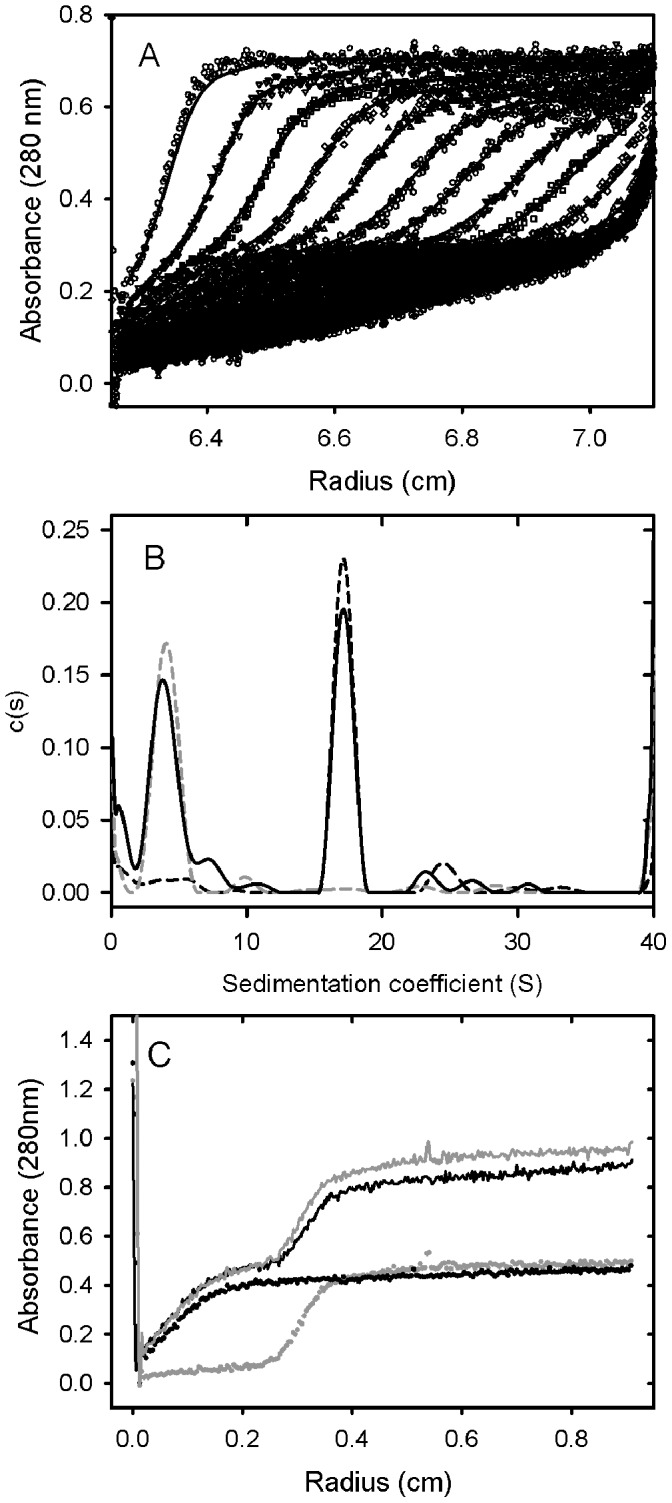
Sedimentation velocity analysis of a mixture of PAT-SM6 and GRP78. A. 0.4 mg/mL of PAT-SM6 and 0.4 mg/mL of GRP78 were incubated together and centrifuged at 28,000 rpm. The resulting sedimenting absorbance boundaries were monitored at 280 nm and representative data shown as open circles. Fits to the experimental data using a c(S) sedimentation model are shown as solid lines. B**.** Corresponding c(S) size distribution plots for the data presented in (A) for PAT-SM6 (black dashed line), GRP78 (grey dashed line) and the mixture of the two proteins (solid line). C. Radial distributions obtained after centrifugation at 28,000 rpm for 72 min for PAT-SM6 (0.4 µM; grey circles), GRP78 (10 µM; black circles) and mixture of PAT-SM6 (0.4 µM) and GRP78 (10 µM, solid line). The solid grey line is the sum of the radial distributions obtained for PAT-SM6 and GRP78 alone, assuming no interaction.

Sedimentation velocity data for purified GRP78 showed a single major sedimenting boundary (Figure 1). Analysis of this data assuming a continuous size distribution of non-interacting species indicated a dominant species characterized by a sedimentation coefficient, frictional coefficient and molecular mass consistent with the presence of GRP78 monomer (Table 1). In addition to this major species, the data identified a concentration-dependent larger species (s20, w of approximately 7.2 S) attributed to the presence of a dimer in slow equilibrium with the predominant monomeric species.

SAXS data for the GRP78 are shown in Figure 2A. The Guinier plot from these data (Figure 2B) was linear at low *q* (where *q*.*Rg* <1.3) and the radius of gyration (*R_g_*) obtained from the slope was 45.5±2.2 Å. The mass of the GRP78 monomer was estimated from the area under the Kratky plot (Figure 2C) to be 89.5 kDa, which is 22% higher than the theoretical value of 73.2 kDa. Given that the in-line gel filtration chromatography allowed us to eliminate the scattering contribution from any dimer, this analysis gives a monomeric protein density of 0.85 g.cm^−1^, which is indicative of the diffuse electron density of a highly flexible molecule [28]. Inspection of the Porod-Debye plots (Figure 2D & 2E) showed the GRP78 SAXS data did not plateau as *I*(*q*).*q* ^4^ vs. *q* ^4^, but did plateau as *I*(*q*).*q* ^3^ vs. *q* ^3^, which further supports the suggestion that GRP78 is highly flexible, but contains significant folded structure [28]. As a control, similar analysis was conducted on the SAXS data recorded from a compact globular protein, glucose isomerase. These analyses showed a plateau in both *I*(*q*).*q* ^3^ vs. *q* ^3^ and *I*(*q*).*q* ^4^ vs. *q* ^4^ plots and gave a density of 1.32 g.cm^−1^, which is in excellent agreement with previous studies of this molecule [28].

**Figure 6:**
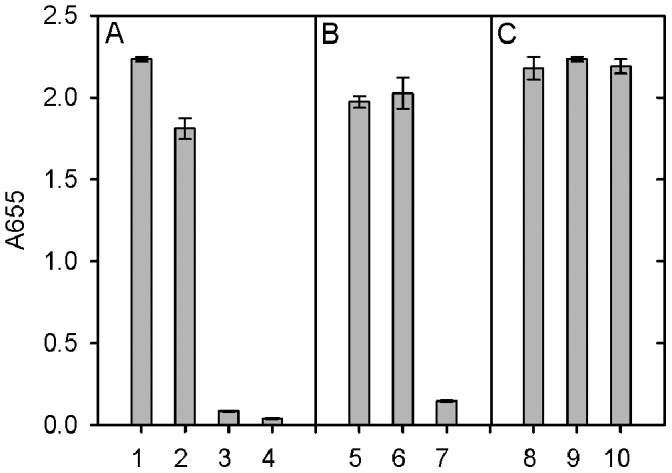
ELISA analysis of the interaction between PAT-SM6 and GRP78. The concentration of GRP78 used to coat the wells was 30µg/mL. A: Primary antibodies used were PAT-SM6 (lanes 1 and 3), anti-GRP78 (lane 2) and control isotype IgM antibody (lane 4). In lane 3 the initial coating step with GRP78 was omitted. B: Primary antibody used was PAT-SM6. Incubations were performed in 20 mM sodium phosphate buffer with no added NaCl (Lane 5), 140 mM NaCl (lane 6) and 500 mM NaCl (lane 7). C: Primary antibody used was PAT-SM6. Incubations were performed in 20 mM sodium phosphate buffer, 140 mM NaCl at pH values of 6 (lane 8), 7.4 (lane 9) and 8 (lane 10).

*Ab initio* shape modelling of GRP78 was performed following estimation of the particle vector distance distribution function *P*(*r*) by indirect Fourier transformation. The maximum dimension (*D_max_*) from *P*(*r*) analysis was estimated to be 134 Å. Figure 2F shows the resulting average volume filtered SAXS model superimposed on the NMR refined full length structure of DnaK [26]. The overall shape and average relative orientation of the N-domain and C-domain of the GRP78 is consistent with the NMR data for DnaK, further establishing the predominantly monomeric nature and correct folding of the bacterial expressed human GRP78 product.

### Characterisation of Purified PAT-SM6

The structure and solution behaviour of purified PAT-SM6 was also characterized. CD spectra for PAT-SM6 revealed a single minimum at approximately 215 nm, suggesting a predominantly β-sheet secondary structure (Figure 3). The results compare favourably with previous CD studies on the secondary structure of IgM antibodies [29]. Analysis of the CD data for PAT-SM6 indicated secondary structure composed of approximately 3.8% á-helices, 51% â-strands, 7.7% turns and 35.8% unordered structure. Sedimentation velocity data for PAT-SM6 showed a single major boundary (Figure 3). Analysis of the data, assuming a continuous sedimentation coefficient distribution of non-interaction species, revealed the presence of one major and one minor population characterised by average sedimentation coefficients of 17 S and 24 S, respectively (Table 1). These results are consistent with the presence of monomeric and dimeric IgM species [30]. The best-fit value obtained for the frictional ratio, f/fo, was used to analyse the overall shape of the PAT-SM6 monomer. The data indicate an asymmetric shape corresponding to an oblate ellipsoid with the parameters listed in Table 1. Sedimentation velocity data was also obtained for a hexameric PAT-SM6 derivative, PAT-SM6-hex, lacking a J chain [11]. Size-distribution analysis of the data indicate a major sedimenting species characterized by s20, w and f/fo values of 17.6 S and 1.95, respectively.

**Figure 7:**
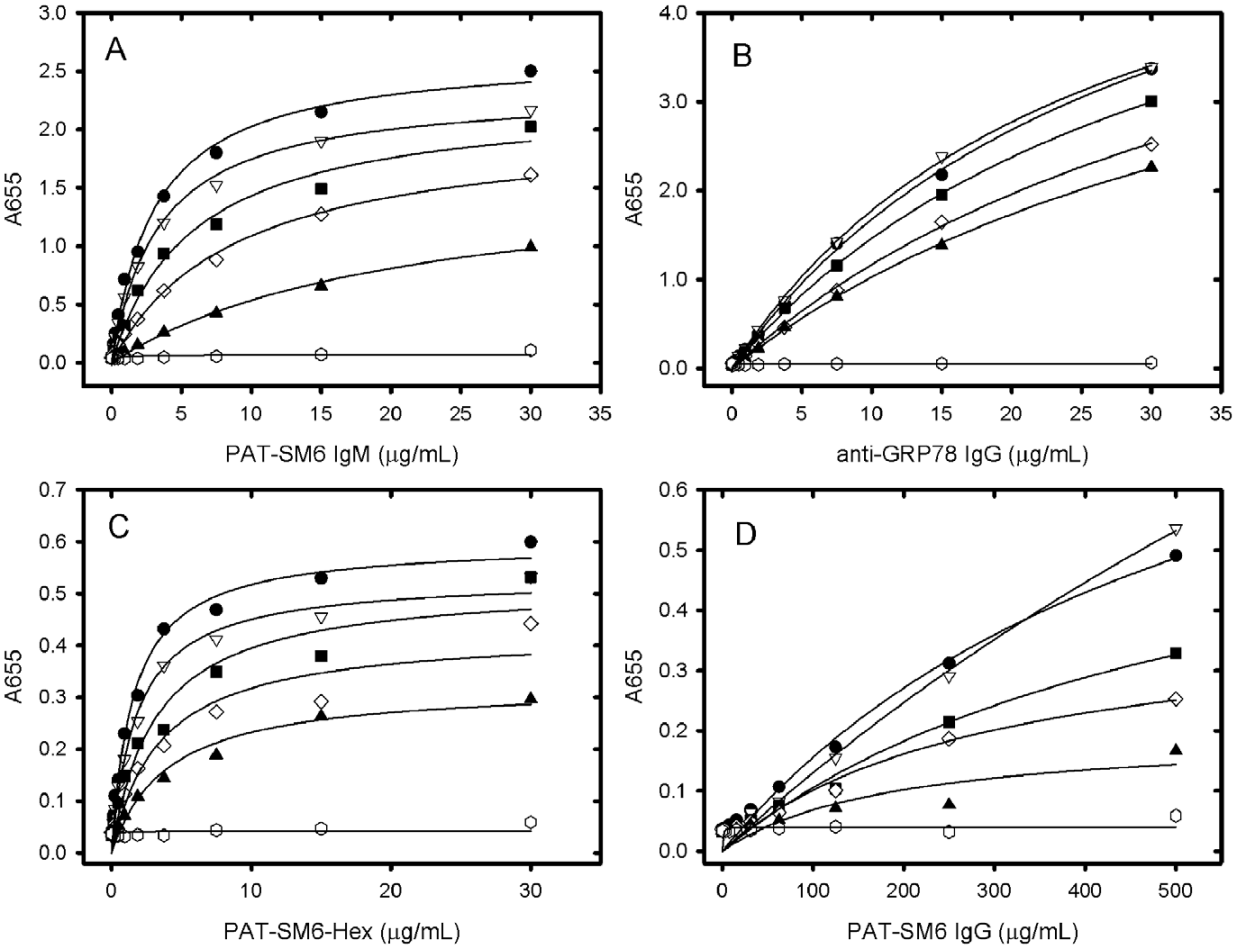
Effect of GRP78 coating concentration on the binding of PAT-SM6 to immobilised GRP78. The primary antibodies used were: A. PAT-SM6; B. polyclonal anti-GRP78; C. PAT-SM6-hex and D. PAT-SM6 IgG. The GRP78 coating concentrations were 30 µg/mL (closed circles), 15 µg/mL (open inverted triangles), 7.5 µg/mL (closed squares), 3.75 µg/mL (open diamonds), 1.88 µg/mL (closed triangles), 0 µg/mL (open circles). The solid lines are best-fit lines calculated for a simple binding isotherm.

**Figure 8:**
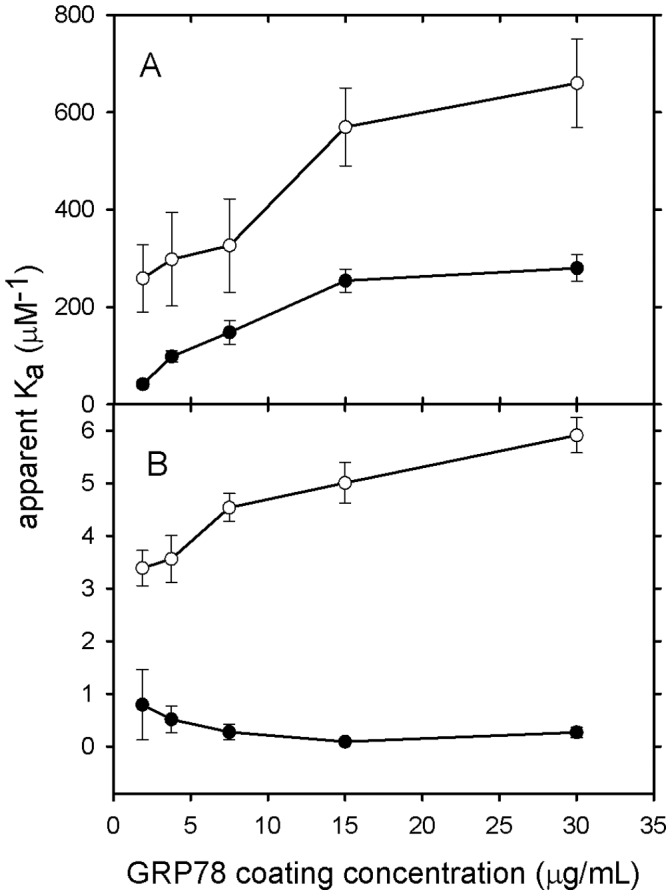
Apparent binding constants (K_a_) obtained from the analysis of the data in **Figure 7** obtained as a function of GRP78 loading concentration. A. Binding constants for PAT-SM6 (closed circles) and PAT-SM6-hex (open circles). B. Binding constants for PAT-SM6 IgG (closed circles) and anti-GRP78 (open circles).

SAXS data for PAT-SM6 are presented in Figure 4A. The Guinier plot (Figure 4B) is linear at low *q* (*q*.*R_g_* <1.3) and the *R_g_* obtained from the slope was 122.0±1.4 Å. The *D_max_* was estimated from *P*(*r*) analysis to be ∼4.2 nm and the molecular mass derived from the area under the Kratky plot (Figure 4C) was 1.06×10^6^ Da. These estimates of mass, *R_g_* and *D_max_* values for PAT-SM6 are in good agreement with those from previous SAXS studies of IgM [31]. Again, the presence of a plateau in the plot of *I*(*q*).*q* ^3^ vs. *q* ^3^ (Figure 4D) and lack of a plateau in the *I*(*q*).*q* ^4^ vs. *q* ^4^ plot (Figure 4E) indicated a folded structure with significant flexibility [28]. *Ab initio* shape modelling of PAT-SM6 did not converge on a physically realistic shape without the imposition of explicit symmetry constraints. Rather than arbitrarily imposing five-fold symmetry, the goodness of fit of the theoretical SAXS profiles of the models with different symmetries (P2, P3, P4, P5 and P6) to the SAXS data were compared [24]. Five-fold symmetry yielded the best fit, which was significantly better that the fits with four-fold or six-fold symmetry (*P*(*F*) values of 0.036 and 0.004, respectively). The average SAXS shape model of the PAT-SM6 monomer with five-fold symmetry agreed well with the overall features characterised in previous electron microscopy, SAXS and homology modelling studies of IgM [31], [32], [33]. Multi-chain rigid body refinement against the SAXS data was performed using Fab fragments flexibly linked to Fc fragments based on IgE [34], as per recent EM modelling [33]. P5 symmetry was imposed and the relative orientation of Fc fragments was constrained by distance restraints corresponding to the inter-subunit disulphide bonds [35]. The SAXS *ab initio* shape model is shown overlaid on the rigid body refined SAXS model (Figure 4F). Both SAXS models suggest asymmetry in the relative positioning of the two Fab fragments within each of the five heavy chain-light chain pairs, which is consistent with the EM shape reconstructions [35]. These structural studies confirm the relative homogeneity of the PAT-SM6 preparation and the suitability for solution studies on the interactions with specific antigens.

**Figure 9:**
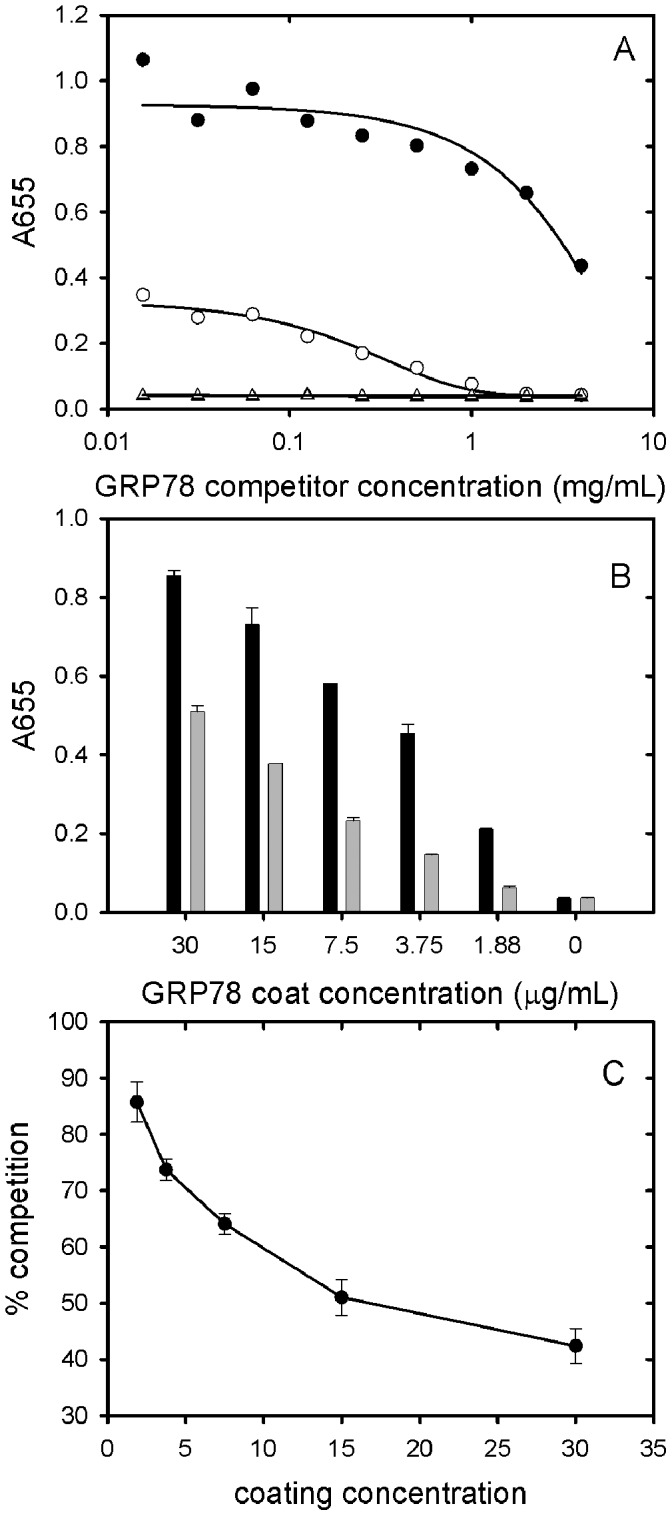
Competition ELISA data obtained using soluble GRP78 as a competitor. A. The concentration of GRP78 used to coat the wells was 7.5 µg/mL. The primary antibodies used were PAT-SM6 (5 µg/ml, closed circles) and polyclonal anti-GRP78 (open circles). Results are also presented for control experiments omitting GRP78 coat using as primary antibodies PAT-SM6 (5 mg/ml, closed triangles) or anti-GRP78 (open squares). B: The binding of PAT-SM6 (5 µg/ml) to immobilized GRP78 at different GRP78 coating concentrations in the absence (black bars) or presence (grey bars) of soluble GRP78 (4 mg/ml). C: Soluble GRP78-induced inhibition of PAT-SM6 binding to immobilised GRP78 at different GRP78 coating concentrations. The concentrations of PAT-SM6 and soluble GRP78 used were 5 µg/ml and 4 mg/ml respectively.

### The Interaction of PAT-SM6 with GRP78 in Solution

Sedimentation velocity analysis was used to investigate the interaction of PAT-SM6 and of GRP78. The radial scans in Figure 5A, obtained for a mixture of PAT-SM6 (0.4 µM) and GRP78 (10 µM), show a slow and a fast moving boundaries corresponding approximately to the boundary velocities obtained for separate samples of GRP78 and PAT-SM6, respectively (Figures 2 and 4). Continuous size distribution analysis of the data obtained for the PAT-SM6-GRP78 mixture confirmed two major populations characterized by average sedimentation coefficients similar to the average values for GRP78 and PAT-SM6. Analysis of the area under the population corresponding to PAT-SM6 indicated a small decrease in area of approximately 10%, consistent with the loss of material. This loss was attributed to the formation and rapid depletion of large antibody-antigen complexes. Further evidence for the loss of material due to complex formation is provided by a comparison of the radial scans taken at a single time point for PAT-SM6 alone, GRP78 alone and the mixture of PAT-SM6 and of GRP78. The results show that the plateau value for the PAT-SM6-GRP78 mixture is lower than the combined optical density scans for the separate components, indicative of complex formation. A difficulty in the analysis of data for weak reversible complexes is the dynamic nature of the interactions and the complexity of fitting the data to specific models [36]. Nevertheless, the data in Figure 5C indicate the loss of only a small amount of PAT-SM6 as large complexes, corresponding to approximately 10% of PAT-SM6. Since the total concentration of PAT-SM6 and GRP78 used in these experiments was 0.4 µM and 10 µM, respectively, this estimate for the proportion of complex yield an estimate for the dissociation constant, assuming a 1∶1 complex, of approximately 90 µM. This relatively low binding affinity is typical of IgM-antigen interactions compared to the interactions between affinity maturated IgG antibodies and their cognate antigens [37].

### ELISA Analysis of the Interaction between PAT-SM6 and GRP78

The interaction of PAT-SM6 with GRP78 immobilised on a microtiter plate was analysed using an ELISA assay. The results in Figure 6 show a strong positive signal using either PAT-SM6 or anti-GRP78 as the primary antibody. Control experiments omitting the initial GRP78 coating procedure or using an IgM isotype control antibody gave a signal that was close to background. The magnitude of the positive signal for the interaction of PAT-SM6 with immobilised GRP78 was similar for samples incubated in 20 mM sodium phosphate buffer, pH 7.4 compared to PAT-SM6 samples in PBS while the signal was significantly diminished when the NaCl concentration was increased to 500 mM. The binding of PAT-SM6 to immobilised GRP78 was similar for incubations performed at pH values of 6, 7.4 and 8.

In view of the relatively strong signal observed for the interaction of PAT-SM6 with GRP78 in ELISA assays (Figure 6) compared to the relatively weak interaction deduced from sedimentation velocity studies (Figure 5B and C) additional experiments were performed to determine the strength of the interactions observed under ELISA conditions. The results in Figure 7A show the ELISA signal determined as a function PAT-SM6 concentration for different coating concentrations of GRP78. The results show typical saturations curves where the magnitude of the ELISA signal at saturation increases systematically as the coating concentration of GRP78 PAT-SM6 is increased. Also shown in Figure 7 are control experiments using different concentrations of anti-GRP78, PAT-SM6-hex and PAT-SM6 IgG. In each case there is a systematic increase in the magnitude of the saturation curves with increased GRP78 coating concentration. A noticeable difference, however, is that the concentration of the primary antibody required for half maximum saturation decreases for PAT-SM6 and PAT-SM6-hex as the coating concentration increases. This effect is not evident when anti-GRP78 or PAT-SM6 IgG are used as the primary antibodies. To quantitate this effect, the saturation curves were fitted to a simple binding isotherm to estimate the apparent binding constants. The results in Figure 8A show that the measured binding constants for PAT-SM6 binding to immobilized GRP78 increase approximately 10-fold over the GRP78 coating concentration range studied. Similarly, there is a significant increase in the binding constant determined for PAT-SM6-hex while there are only small changes in the binding constants for either anti-GRP78 or PAT-SM6 IgG (Figure 8B). The dependence of the binding constant for PAT-SM6 on GRP78 coating concentration implies that the interaction depends on the clustered nature of the GRP78 coat and that the interaction is driven by an avidity affect arising from the high valency of both PAT-SM6 and PAT-SM6-hex. The binding constant of approximately 250 µM^−1^ obtained for the binding of PAT-SM6 to GRP78 at a GRP78 coating of 30 µg/ml, converts to a dissociation constant of approximately 4 nM, a significantly lower value than the dissociation constant of 90 µM deduced from the sedimentation velocity studies of the solution interaction between PAT-SM6 and GRP78 (Figure 5). In contrast, the apparent binding constant for PAT-SM6 IgG binding to immobilized GRP78 is much lower, yielding an estimate for the dissociation constant of approximately 3 µM. These comparisons between the binding of PAT-SM6 to soluble GRP78 and to immobilised GRP78 and the difference in binding affinities observed for PAT-SM6 compared to PAT-SM6 IgG implies that the strength of the interaction with immobilized GRP78 is driven by the multivalent nature of PAT-SM6 and the ability to bind simultaneously to multiple molecules of GRP78 clustered on the microtiter plate. Additional evidence for the multivalent nature of the interaction of GRP78 with PAT-SM6 is provided by competition ELISA experiments (Figure 9). The results show that substantially higher concentrations of competitor GRP78 are required for half maximum inhibition of PAT-SM6 binding compared to anti-GRP78 binding to immobilized GRP78. Additional competition ELISA experiments showed that the degree of soluble GRP78-induced inhibition of PAT-SM6 binding varied inversely with the concentration of GRP78 used to coat the plate. These results suggest that the multivalent binding of PAT-SM6 to immobilised GRP78 is determined by the degree of GRP78 clustering on the plate such that inhibition by competitor GRP78 more effective at lower coating concentration where the extent of clustering is reduced.

## Discussion

Sedimentation velocity and SAXS analysis of purified PAT-SM6 revealed a single major species with a pentameric structure characteristic of the IgM class of antibodies. These natural antibodies form part of the first line of defence and typically show low antigen specificity with the ability to bind more than one type of antigen [38]. PAT-SM6 was originally isolated from a gastric cancer patient and shown to bind to cell membrane-associated GRP78 in tumour cells [9] as well as oxidized plasma low density lipoproteins [8]. The present study shows that the interaction of purified PAT-SM6 with GRP78 is mediated through an avidity-based mechanism based on the multivalency of the antibody. Several observations support this conclusion. The interaction of soluble GRP78 and PAT-SM6 analysed by sedimentation velocity revealed a low level of complex formation. The estimate obtained for the dissociation constant of approximately 90 µM, assuming a 1∶1 complex, is comparable to values obtained from sedimentation velocity studies of IgM interactions with complement component C1q which yield values in the 100 µM concentration range [39]. These relatively weak, low affinity interactions appear to be characteristic of IgM-antigen interactions, when these species are free in solution, compared to the more specific, affinity maturated interactions observed for IgG-antigen complexes [37]. In contrast, strong interactions between immobilized GRP78 and PAT-SM6 were observed in ELISA experiments. Furthermore, the interactions between PAT-SM6 and GRP78 detected in ELISA experiments were dependent on the GRP78 coating concentration used. This dependence on coating concentration was not observed for a PAT-SM6 IgG derivative or a polyclonal anti-GRP78 IgG control antibody. In addition, the ability of soluble GRP78 to compete in the interaction between PAT-SM6 and immobilised GRP78 was more effective at lower GRP78 coating concentrations. These observations are consistent with a binding model whereby the strength of the interaction between PAT-SM6 and GRP78 depends on the simultaneous binding of multiple sites on PAT-SM6 to GRP78 clustered on the microtiter plate. Such clustering may also be characteristic of GRP78 over-expressed on the cell membrane of tumour cells accounting for the strong binding of PAT-SM6 to tumour cells and the accompanying high-level of apoptosis [9].

The present study used recombinant GRP78 that was expressed and purified from *E. coli.* Sedimentation velocity and SAXS analysis indicated the purified product was correctly folded while mass spectrometry confirmed the lack of glycosylation that occurs during mammalian GRP78 expression. Both glycosylated and non-glycosylated forms of GRP78 have been detected on the surface of cancer cells [9] with experimental evidence that PAT-SM6 interacts more strongly with O-glycosylated forms of GRP78 which are specific for malignant cells. The present results indicate that PAT-SM6 interacts with high avidity with non-glycosylated GRP78 suggesting that at least part of the apoptotic affect is independent of the state of GRP78 glycosylation. It remains a possibility however, that glycosylated forms of GRP78 may bind with even higher affinity to PAT-SM6 leading to more effective induction of cell death. Further studies are needed to determine whether the ability of PAT-SM6 to induce tumor cell apoptosis is dependent on the glycosylation status of tumor cell surface GRP78.
